# Transient Global Amnesia: An Electrophysiological Disorder Based on Cortical Spreading Depression—Transient Global Amnesia Model

**DOI:** 10.3389/fnhum.2020.602496

**Published:** 2020-12-08

**Authors:** Xuejiao Ding, Dantao Peng

**Affiliations:** ^1^Department of Neurology, China-Japan Friendship Hospital, Beijing, China; ^2^Graduate School of Peking Union Medical College, Chinese Academy of Medical Sciences, Beijing, China

**Keywords:** transient global amnesia, spreading depolarization, cortical spreading depression, hippocampus, stress, locus coeruleus, glutamate, glia

## Abstract

Transient global amnesia (TGA) is a benign memory disorder with etiologies that have been debated for a long time. The prevalence of stressful events before a TGA attack makes it hard to overlook these precipitating factors, given that stress has the potential to organically effect the brain. Cortical spreading depression (CSD) was proposed as a possible cause decades ago. Being a regional phenomenon, CSD seems to affect every aspect of the micro-mechanism in maintaining the homeostasis of the central nervous system (CNS). Corresponding evidence regarding hemodynamic and morphological changes from TGA and CSD have been accumulated separately, but the resemblance between the two has not been systematically explored so far, which is surprising especially considering that CSD had been confirmed to cause secondary damage in the human brain. Thus, by deeply delving into the anatomic and electrophysiological properties of the CNS, the CSD-TGA model may render insights into the basic pathophysiology behind the façade of the enigmatic clinical presentation.

## Introduction

Transient global amnesia (TGA) is an episodic memory disorder that affects the hippocampus (Eustache et al., 1999). It typically occurs in middle-aged to elderly populations (Arena and Rabinstein, 2015). A large proportion of TGA cases are preceded by stressful events which may be emotional or physical (Fisher, 1982). Gender differences in these precipitating incidents vary according to different studies, but men tend to have physical stress, while women are more likely to experience emotional turmoil prior to attacks (Quinette et al., 2006). Recurrence is relatively low but it may be increased in those with certain prior conditions such as head injury or psychological disorders (Tynas and Panegyres, 2020).

The chief complaint of TGA patients is anterograde amnesia, which lasts up to 24 h without focal neurological signs and symptoms (Romero et al., 2013). Anterograde amnesia manifests as an inability to acquire new information, and patients typically repeat the same question despite being repetitively provided with the answer (Agosti et al., 2008). Other features include retrograde amnesia, mainly confined to recent events that happened prior to TGA, and/or vegetative symptoms such as headache or nausea (Arena and Rabinstein, 2015). An anterograde memory gap is seldom recovered, but certain retrograde components may be regained (Eustache et al., 1999). Several etiologies have been proposed, such as migraine, epilepsy, ischemia, venous congestion, and glutamate toxicity (Romero et al., 2013; Ogawa et al., 2018; Han et al., 2019). But none of these disorders could sufficiently explain the disorder in its entirety. As another postulated cause of TGA, cortical spreading depression (CSD) has been found to exert a detrimental impact on patients with critical conditions such as traumatic brain injury (Dreier et al., 2017). It was first proposed as a possible etiology of TGA in 1986 (Olesen and Jørgensen, 1986). Research findings on both TGA and CSD have accumulated over the past few decades. These two distinct conditions seem to share many overlapping domains. Nevertheless, the relationship between the two has not been systematically delineated so far. In this article, mechanisms underpinning a stress-induced locus coeruleus-noradrenaline (LC-NE)-related CSD–TGA model will be explored for the first time, demystifying this perplexing disorder and bridging the gap between clinical presentation and the possible electrophysiological alterations on a holistic perspective.

## Cortical Spreading Depression

CSD or spreading depolarization (SD) perturbs the homeostasis of neurons and glial cells by increasing the levels of extracellular K^+^/glutamate and intracellular Ca^2+^/Na^+^/water content, resulting in cell swelling and unresponsive cells (Dreier, 2011). SD has been observed in the penumbra in patients with metabolically stressed brain tissue (Mayevsky et al., 1996). Under experimental conditions, CSD can be triggered in normal brain tissue by various methods including direct application of KCl/glutamate or mechanical/electrical stimulation (Olesen and Jørgensen, 1986; Zandt et al., 2013). By depolarizing cells to various degrees, CSD depresses the spontaneous activity of brain cells and induces hypoxic stress on brain metabolism (Pietrobon and Moskowitz, 2014).

Upon neuronal activation, the neurovascular unit (NVU) reacts by dilating the cerebral vasculature in order to meet the increased metabolic needs of the brain (Iadecola, 2017). This phenomenon is termed neurovascular coupling (NVC) and is coordinated by various factors such as vasoactive substances released from activated neurons and glial cells, contractible pericytes, endothelial cells mediating retrograde vasodilatation, and astrocytic end-feet wrapping around the cerebral vasculature (Henneberger et al., 2010; Itoh and Suzuki, 2012; Mishra, 2017; Iadecola, 2017). Complex electrophysiological changes during CSD result in unresponsive cells and neurovascular (NV) uncoupling which manifests as decreased regional cerebral blood flow (rCBF) by vascular unresponsiveness or even vasoconstriction after initial depolarization-induced hyperemia, with long-lasting hypoperfusion (Leao, 1944; Lauritzen, 1984; Chang et al., 2010; Wainsztein and Rodríguez Lucci, 2018).

## CSD–TGA Model

Since a large proportion of patients experience emotional or physical exertion prior to symptoms, stress, as an initiating factor, may trigger the onset of TGA (Griebe et al., 2019). Stress-induced memory deficits are not rare considering that psychogenic factors are more prominent in dissociative amnesia (Thomas-Antérion, 2017). Focal retrograde amnesia (FRA) features more profound memory deficits in younger populations, while the precipitating events are more likely to be organic, such as traumatic incidents (Stracciari et al., 2008). Hence, TGA and FRA may share similar pathological features with varying intensities of insults and different degrees of hippocampal vulnerability.

Stressful events activate the central nervous system (CNS) by working on the locus coeruleus-noradrenaline (LC-NE) system (Berridge and Waterhouse, 2003). The LC is a pontine nucleus rich in noradrenergic neurons which project extensively throughout the brain, with dense projections to the entry zone of the hippocampus-dentate gyrus (DG; Samuels and Szabadi, 2008; Mancall and Brock, 2011). Upon receiving stimulation from the LC-NE system, glutamatergic granule cells in the DG amplify their impact by pairing with multiple postsynaptic pyramidal cells and interneurons in the cornu ammonis (CA) which is also rich in glutamatergic neurons (Okubo and Iino, 2011). Being an effective CSD initiator, the quantum release of glutamate, accompanied by typical CSD constellations of substrates such as K^+^ and Ca^2+^, would trigger CSD once it reaches the threshold concentration (Pietrobon and Moskowitz, 2014). Thus, wide-spread existence of LC-NE and glutamate neurons give the CSD-TGA model an anatomical basis.

In TGA, stressful events may trigger physiological cascades similar to those of CSD. Upon exertion, a volume release of stress-related neurotransmitters including NE induces the hyperexcitability of glutamatergic neurons in the hippocampus. Depolarized neurons, interneurons, and nearby glial cells result in the cascading mobilization of CSD initiators such as glutamate, K^+^, and Ca^2+^. Once a certain limit is exceeded, CSD would be initiated. Excess glutamate reaches beyond the postsynaptic receptors through volume transmission (VT), spreading electrophysiological cascades to nearby regions (Okubo and Iino, 2011). Glutamate positive-feedback further places neurons at a risk of CSD especially when buffered from astrocytes failure (Xu et al., 2007). Normally, functional units on astrocytes such as the K^+^ channels or glutamate transporters would uptake these CSD initiators as well as other substances to maintain a biochemical equilibrium in extracellular space (Kandel et al., 2013). Apart from facilitating uptake, gap junctions (GJs) also help maintain local homeostasis by transporting solutes between cells without overwhelming them (Epifantseva and Shaw, 2019). Not considered to be major players in CSD, astrocytes facilitate electrical signaling through IP_3_-mediated Ca^2+^ waves *via* GJs, featuring glutamate induction and K^+^ efflux (Goldberg et al., 2010; Tamura et al., 2011). Also, if Ca^2+^ levels reach a certain threshold, GJs shut down (Kandel et al., 2013). Disrupted astrocytic syncytium further compromises astrocytic buffering, facilitating the accumulation of glutamate/K^+^ and aggravating cell swelling. Both glutamate and K^+^ may induce vessel constriction once their concentrations reach a certain threshold (Seidel et al., 2016; Borgdorff, 2018). Therefore, the direct vasoactive effects of chemical substrates are superseded by CSD-induced NV uncoupling. Being a neuroexcitatory chemical, glutamate may also cause a direct toxic effect on brain cells. But, considering the naivety of TGA lesions, there seems to be certain limitations on the impact of the insult, which renders glutamate toxicity as the cause of TGA unlikely because it usually leads to irreversible cell death (Landolt et al., 1998).

The reduced release of nitric oxide (NO) caused by CSD compromises its permissive role in vasodilating reactions to CO_2_ during TGA attack (Scheckenbach et al., 2006; Hunter, 2011). The need to repolarize coupled with vasoconstriction increases the metabolic stress which manifest itself as increased lactate concentration on MRS in the hippocampus in TGA patients and in those with SD-induced secondary ischemia (Bartsch and Deuschl, 2010; Yuzawa et al., 2012; Dreier et al., 2017). Contrary to initial depolarization-paired hyperemia and the increased cerebral metabolic rate of oxygen (CMRO_2_), CSD-induced suppression of spontaneous neuronal activity and oligemia lead to reduced CMRO_2_ in mice (Shetty et al., 2012; Yuzawa et al., 2012; Pietrobon and Moskowitz, 2014). In TGA, decreased metabolic rate and CMRO_2_ have been observed in memory-related areas on a PET scan (Baron et al., 1994; Eustache et al., 2000).

CSD also has temporal characteristics regarding its phasic progression. NV uncoupling causes contradictory hemodynamics with hypoperfusion being more prominent on SPECT during TGA attack (Tanabe et al., 1991; Fabricius and Lauritzen, 1993). Osmotic imbalances result in swollen cells and narrowed interstitial space, leaving abnormal restricted diffusion on an MRI among TGA patients (Strupp et al., 1998; Dreier, 2011). Also, local cell density, topography of the gyrus, and vascular structures confine the progression of CSD, resulting in regional DWI lesions (Van Harreveld and Fifková, 1970; McBain et al., 1990; Bartsch and Deuschl, 2010; Borgdorff, 2018).

Complex ion mobility underpins the slow progression of CSD (Van Harreveld and Fifková, 1970). This feature results in the delayed appearance of abnormal diffusion restrictions and slow recovery from morphological alterations. As observed in TGA, electrophysiological abnormalities such as reduced fMRI signal or hypoperfusion on PET in memory networks often appear during the acute phase, before the cytotoxic oedema shows on DWI (Tanabe et al., 1991; Peer et al., 2014). In this regard, the duration of clinical presentation is typically less than 12 h, whereas positive DWI findings may manifest 24–48 h after symptom onset and persist for 7–10 days (Bartsch and Deuschl, 2010).

It has been proposed that bilateral hippocampal deficits might be common findings if timely radiological examinations are obtained, since amnesiac symptoms such as TGA could only be caused by bilateral malfunctions (Peer et al., 2014; Arena and Rabinstein, 2015). It is unlikely that both hippocampi are simultaneously loaded with an above-threshold level of CSD initiators every time TGA happens. But what could be temporally contiguous enough to cause bilateral hippocampal malfunction leading to TGA? The two hemispheres of the human brain coordinate activity *via* the corpus callosum, a white matter tract connecting the two hemispheres and relaying reciprocal information (Mancall and Brock, 2011). Activation and coordination among neural networks require effective afferent input, and the lack of which may depress its downstream connections. In the original findings of Leao, there is a latency between depression in the CSD-targeted region and the detectable electrical activities of the corresponding site on the contralateral hemisphere. It may not be due to the actual crossing of the original CSD as Leao proposed, since CSD is not able to spread along the white matter, but may be from the lack of effective input from the original lesions. As is the case in one TGA study, decreased activation of memory-related networks were detected bilaterally in all patients, but DWI lesions were only observed in the hippocampus unilaterally (Peer et al., 2014). The side with low activation may be due to an original attack with comparatively less intensity compared with the other side, or it may also be due to a smaller signal input from the contralateral side, or both. Therefore, the malfunctions in memory networks mentioned above suggest that electrophysiological pathology underpins TGA pathology. But impaired neuronal connectivity is not specific to TGA. Any type of lesion interfering with the functionality of upstream signaling is likely to produce similar phenomena. For example, thalamic stroke and transient epileptic amnesia also impair the efficacy of memory circuits, displaying similar clinical presentations like TGA (Giannantoni et al., 2015; Takeda et al., 2020).

## Amnesia

The chief complaint of TGA is amnesia, which consists of anterograde and/or retrograde components. Suppressed neurons, sustained by energy deprivation, may be unable to encode and/or store new information by impaired activity-dependent memory formation, resulting in anterograde amnesia.

Retrograde amnesia mainly comprises ecmnesia (confined to recent events) and may be subsequently recovered (Eustache et al., 1999). This may reflect a retrieval problem considering that decreased activity in the prefrontal cortex is involved in poor memory retrieval (Zidda et al., 2019). After the TGA attack, older memories are recovered more efficiently than recent ones (Peer et al., 2014). Why is this? Newly formed memories typically undergo consolidation and are highly unstable, indicating a consolidation gradient (Bisaz et al., 2014). Long-term potentiation (LTP) and long-term depression (LTD), by way of gradient excitability and coactivation of both pre- and post-synapses, contribute to synaptic plasticity (SP) and memory consolidation in the hippocampus (Hebb, 2002; Feldman, 2012; Nicholls et al., 2012). Therefore, neuronal silencing during CSD may impair plasticity by dissociating Hebbian synaptic coactivation and by suspending on-going pulses from LTP/LTD. It is observed that theta waves, which facilitate LTP, are altered (slow or decreased) in TGA/migraine patients as well as CSD animal models (Ogunyemi, 1995; Park et al., 2016; Lourenço et al., 2017). On a biochemical level, CSD interferes with LTP by manipulating the efficacy of glutamate receptors such as GluN2A/B and AMPA (Maneepark et al., 2012; Hansrivijit et al., 2015). CSD may also impair memory consolidation by attenuating nitric oxide (NO) production since NO facilitates LTP and LTD (Daniel et al., 1993; Zhang et al., 2002; Bon and Garthwaite, 2003).

The maintenance of synaptic connections underlying formed memories requires complex biochemical interactions and distinct neuronal activities other than LTP and LTD such as depotentiation (Neves et al., 2008). Considering the drastic electrophysiological changes produced by CSD, other biological processes regarding memory formation may also be influenced. Therefore, retrograde amnesia in TGA may not be confined to retrieval deficits, but also touches upon active processes of neural plasticity.

## Recovery

Recovery from CSD is not a passive process, but an active coordination among multiple biochemical dimensions. During CSD, since only a small amount of ATPase is affected, additional functional units are able to be recruited to re-establish homeostasis (Drenckhahn et al., 2012; Pietrobon and Moskowitz, 2014). Also, neurotrophic and/or anti-ischemic gene expression, including glial fibrillary acidic protein (GFAP) and i/nNOS, are induced during SD in ischemic tissue (Matsushima et al., 1996; Scheckenbach et al., 2006). When the hippocampus is devoid of energy supply, brain cells may use other metabolites such as glutamine as alternative fuels (Amaral et al., 2011; Bernier et al., 2020). Therefore, brain cells harness self-survival mechanisms to try to regain homeostasis even under electrical depression, which gives them the opportunity to bounce back to normal. Especially when the targeted tissue has a relatively adequate energy reserve, this coordination can be more efficient and lesion reversibility is justified.

With the accumulation of metabolic waste in ISF, local vasculature begins to dilate, bringing oxygenated blood to hypoxic tissue, allowing for the repolarization of brain cells (Grafstein, 1956; Dreier, 2011). Local homeostasis is slowly achieved mainly by recovered functional units (pumps and channels; Dreier, 2011). Metabolic waste is swept away by the glymphatic system. In healthy brain tissue, depressed neuronal activity will recover the moment CSD passes, whereas in compromised brain tissue the electrical silencing may linger (Lauritzen et al., 2010). As in TGA, prolonged hemodynamic disturbances persist after the amnesiac symptoms recover, accompanying the delayed DWI signals (Bartsch and Deuschl, 2010). This lasting NV uncoupling indicates that a TGA hippocampus may not be in a “healthy” enough condition in the first place to allow for spontaneous electrical recovery.

Additionally, TGA may present with sporadic lesions on DWI, hypoperfusion/functional alterations in the temporal lobe without abnormal diffusion restrictions, or the absence of radiological abnormalities (Lampl et al., 2004; Bartsch and Deuschl, 2010). Damage induced by CSD constitutes a graded continuum with regard to tissue tolerance for insults (Pietrobon and Moskowitz, 2014). Greater insults and more metabolically compromised tissue result in more severe damage (Dreier, 2011; Hartings et al., 2020). In the case of TGA, apart from different timing, various degrees of insult and/or the inherent condition of brain tissue may also contribute to the variability of radiological presentations.

## Vulnerability of The Hippocampus

The reason for selectively targeting the hippocampus is unclear. High metabolic demands and limited energy reservation render the human brain vulnerable to hemodynamic disturbances, especially pyramidal cells in the hippocampus (Dugan and Choi, 1999). The dense distribution of glutamatergic neurons found in the hippocampal formation may add unique neuro-excitability to this region (Tamminga et al., 2012). In humans, graded vulnerability is observed within the hippocampus, with CA1 being the most easily affected area regardless of the nature of the insult (Bartsch et al., 2015). The underlying cause is considered to be greater Ca^2+^ mobility in CA1, which is the hallmark of CSD coupled with glutamate release, and the NMDA receptor is crucial at initiating CSD (Itoh and Suzuki, 2012; Yamashima, 2016). Additionally, when facing a similar level of depolarization, deep gray matter in the brain stem withstands CSD better than cortical areas (Richter et al., 2008). Thus, the unique microanatomical and biochemical composition of the hippocampus may render CA1 a vulnerable target in CSD.

Also, the capability of fending off homeostatic disturbances such as one caused by CSD may be impaired by aging. Distributed on astrocytic endplates in a polarized manner, AQP4 is a crucial component in the glymphatic system for transporting waste from the ISF out of the CNS (Nedergaard, 2013). This distribution pattern declines with age, impairing the efficacy of the glymphatic system (Kress et al., 2014). Also, CSD downregulates the glymphatic system through astrocytic swelling, in combination with an influx of excess CSF into the ISF due to vessel constriction, resulting in waste accumulation and brain oedema (Schain et al., 2017; Mestre et al., 2020). Thus, elderly people with less efficient waste disposal may be less resilient to acute insults such as CSD. Preclinical research has demonstrated diverse AQP4 expression in different areas of the brain, with lower density in the hippocampus (Hubbard et al., 2015). If this pattern is conserved in humans, the hippocampus may face additional vulnerability in combating local metabolic stress induced by CSD.

Not all TGA patients report stressful events, and not all elderly individuals with stressful experiences develop TGA. It has been proposed that certain personality traits of TGA patients may precipitate their hyperactivity to stress, where the prevalence of psychological disorders are more profound among these patients than their healthy controls (Griebe et al., 2019).

Any type of exertion, be it physical or psychological, by targeting the basic structures of the nervous system, resorts to a common pathway to make its biological influence. It may do potential harm if the balance is tipped from evolutionary benefits. It is observed that TGA patients have a HPA hyperreactivity to stress and an elevated level of cortisol, a major player in HPA axis activation during stress (Griebe et al., 2019). Prior stress may also sensitize LC responsiveness to CRH which facilitates the activation of the LC-NE system (Reyes et al., 2006). Thus, stress may create a base for TGA vulnerability by giving CSD initiators a higher capacity of motivation. It has been shown that chronic exposure to stress hormones may predispose the hippocampus to secondary insults (Sapolsky et al., 1990). This predisposition and potential neurodegenerative comorbidities in TGA patients (considering its late age of onset) may increase the vulnerability of these individuals to TGA attacks. Therefore, in these patients, the hippocampus is susceptible to insults which would not usually cause symptoms in their age-specific counterparts.

Long-term exposure to stressful events and cortisol is associated with the small volume of the hippocampus (Starkman et al., 1992). A smaller size of the hippocampus among TGA patients has also been detected (Kim et al., 2017). Thus, the relationship between pre-onset cortisol level with decreased hippocampus size among the TGA population remains to be researched, which may give a certain baseline for their hippocampal wellbeing.

A recently proposed HPA–TGA model explored the role of heightened cortisol secretion in response to stress as an etiological factor of TGA by emphasizing the direct impact of cortisol on brain tissue *via* blood flow (Griebe et al., 2019). Whether or not HPA axis hyperactivity could provide sufficient cortisol to inflict neurotoxicity on brain tissue and why blood cortisol selectively affects the hippocampus requires further exploration, especially considering dilution from blood, efficient astrocytic buffering, and substrates disposal by the glymphatic system.

Apart from a reduced volume of the hippocampus, higher presence of pre-existing hippocampal cavities exists among TGA patients, although these cavities are considered to be naive (Uttner et al., 2011; Park et al., 2015). Although it is believed that hippocampal neurogenesis occurs in the adult human brain and stress could impair this process, there is strong evidence that neurogenesis may not actually occur in adulthood (Warner-Schmidt and Duman, 2006; Sorrells et al., 2018). Either way, any pre-existing damage may place the hippocampus in a more vulnerable position especially in people experiencing long-term stress.

## Discussion

The widespread distribution of the LC-NE system and glutamatergic neurons, the availability of chemical and anatomical factors for CSD induction and propagation, prevalent stress-related conditions and altered stress physiology among TGA patients, and possibly increased hippocampal vulnerability support the CSD–TGA model. An amount of corresponding evidence on hemodynamic and morphological changes from CSD and TGA research, combined with the solid conclusion that CSD could happen in human brain tissue, indicate that CSD may play a role in TGA.

The concept of SD spectrum disorders, including migraine, stroke, traumatic brain injury, and other diseases with secondary tissue damage induced by SD, has been proposed (Dreier et al., 2017). Current technical limitations hinder validation of the relationship between CSD and TGA. Until non-invasive monitoring techniques become available, TGA remains an eligible candidate for SD spectrum disorders.

## Data Availability Statement

The original contributions presented in the study are included in the article, further inquiries can be directed to the corresponding author.

## Author Contributions

XD conceptualized the study and drafted the intellectual contents. DP edited the article. All authors contributed to the article and approved the submitted version.

## Conflict of Interest

The authors declare that the research was conducted in the absence of any commercial or financial relationships that could be construed as a potential conflict of interest.
